# The relationship between grammatical knowledge and reading comprehension: A meta-analysis

**DOI:** 10.3389/fpsyg.2023.1098568

**Published:** 2023-03-13

**Authors:** Haoyuan Zheng, Xuecong Miao, Yang Dong, Daniel-Chongbo Yuan

**Affiliations:** ^1^Faculty of Teacher and Education, Guangzhou Huashang College (廣州華商學院), Guangzhou, China; ^2^Research Center for Overseas Studies and Media Reports on Hainan, Hainan University, Haikou, China; ^3^Faculty of Sociology, Huazhong University of Science and Technology, Wuhan, China; ^4^Department of English, Hainan University, Haikou, China; ^5^Department of Literacy, Hong Kong Metropolitan University, Kowloon, Hong Kong SAR, China

**Keywords:** grammatical knowledge, reading comprehension, reading stage, learning cognitive condition, mental model, meta-analysis

## Abstract

The study aimed to examine the cohesive tie effect on reading comprehension through the grammatical knowledge cognition process. The present meta-analysis examined the correlation between grammatical knowledge and reading comprehension based on empirical results published between 1998 and 2021. This study selected 86 studies with a total of 14,852 readers whose grades were grouped from primary school to university. The results showed that the overall correlation effect size between grammatical knowledge and reading comprehension was large, and the significant interaction effect of the grade group was confirmed through moderator analysis. The results suggested that the grammatical knowledge’s function of the cohesive tie has a transfer effect across different text comprehension scripts.

## Introduction

Reading comprehension refers to an ability to acquire literal or inferential meaning from the given text through the interaction between sentences’ cognition process and situation image construction (Snow, 2002; Cain et al., 2004; Dong et al., 2021, 2022). Regardless of whether it an Eastern or a Western country, reading comprehension plays a vital role in human career development, knowledge base construction, and language ability development and even in printed script-based communication (Carretti et al., 2009; García and Cain, 2014; del Pilar Jiménez et al., 2019). As one kind of foundational knowledge and linguistic skill, grammatical knowledge could influence reading fluency and reading accuracy and further influence reading comprehension through discourse comprehension (e.g., Alderson, 2000, p. 37; Muter et al., 2004; Chik et al., 2012). *Cohesive tie theory* (Cain and Nash, 2011) suggests that grammatical knowledge generates a necessary inference for discourse comprehension through information integration. *Reading stages theory* (Chall, 1996) suggests that grammatical knowledge should have different effects on reading comprehension across different grade groups. Hjetland et al. (2019) and Zhang (2012) reported that the grade of primary and secondary school readers positively moderated the correlation between GK and reading comprehension. Moreover, *cognitive condition theory* (Tagarelli et al., 2011) suggested that the function of grammatical knowledge on the text comprehension process ought to be different between the first-language (L1) and second-language (L2) scripts due to literacy exposure. Zhang and Koda (2018) and Shibasaki et al. (2015) used the structural equation model to report that the GK contributed more to unfamiliar language scripts than to familiar scripts in text comprehension in school-age students. However, the interaction effects between grammatical knowledge and reading comprehension across developed relations (grade group) and literacy exposure (language type) remain unclear. Moreover, extensive studies reported the inconsistent correlations between grammatical knowledge and reading comprehension, and whether the variance could be explained by the interaction effect of reading stages or learning cognitive conditions requires further investigation. Therefore, the current study considered the meta-analytic approach to synthesize empirical studies, investigating the rule of cohesive tie function of grammatical knowledge on text comprehension.

## Literature review

### Grammatical knowledge and reading comprehension

Grammatical knowledge, in general, refers to knowing the rule of grammar application in reading text construction of verb form, word order, and sentence structure (Shiotsu, 2010; Purpura, 2013). Under the framework of the cohesive tie, past studies have confirmed grammatical knowledge as a prerequisite of efficient reading for reading both L1 and L2 texts (Grabe, 2009; Jeon and Yamashita, 2014). This is because grammatical knowledge determines the sentence structure and further impacts reading comprehension through the word function sequence (e.g., Gahl and Garnsey, 2004; Vinyals et al., 2015), which means that grammatical knowledge may assist individual word comprehension or semantic chunk comprehension when readers use the rule of sentence structure for decoding sentence meanings (Tunmer, 1989; Rego and Bryant, 1993); that is, the better the proficiency in grammatical knowledge application, the better the text reading comprehension performance. Readers could take the grammatical rule to assist text comprehension through the indirect contribution of text semantic meaning inference (Rego and Bryant, 1993).

A consensus has been reached on the fact that fully developed grammatical knowledge could allow readers to monitor syntactic information through text comprehension progress. The relationship between grammatical knowledge and reading comprehension is associated with the reader’s ability to generate coherence in the text and monitor meaning acquisition during the reading process (Fender, 2001; Grabe and Stoller, 2012). For example, the grammatical clues offered coherence hints or information to aid readers in constructing text and understanding discourses (Zwaan and Rapp, 2006; Aryadoust and Baghaei, 2016), which indicates that the grammatical knowledge could be regarded as an ability to process syntactic structures and complicated sentences in text comprehension (Zwaan and Rapp, 2006; Aryadoust and Baghaei, 2016).

Previous studies, however, reported various results regarding the role of grammatical knowledge in text reading comprehension, from a strong correlation to a weak correlation. Some studies reported a strong correlation between grammatical knowledge and reading comprehension (Bentin et al., 1990; Muter et al., 2004; Shiotsu and Weir, 2007; Kim and Cho, 2015). Conversely, other empirical surveys and experimental research reported a weak association due to the development effect of lexical inference (Kern, 1989; Kleeck, 2008; Zhang, 2012), knowledge base (Schatschneider et al., 2004; Silva and Cain, 2015; Brimo et al., 2017), and phonology knowledge (Demont and Gombert, 1996; Blackmore and Pratt, 1997; Farnia and Geva, 2013). Therefore, the correlation between grammatical knowledge and text reading comprehension was unclear.

### Potential moderator selection

The current study selected grade group and language type as potential moderators for the following reasons.

#### Grade group

The Reading stages theory (Chall, 1996) claims that readers start learning to read in early primary school to become professional in reading to learn at university. Higher reading stages match more complicated requirements of text reading comprehension and proficiency in grammatical knowledge application. Previous studies also showed various results on the correlation between reading comprehension and grammatical knowledge. For example, Farnia and Geva (2013) reported that younger readers (grade 1) performed better in English grammatical knowledge tests than older readers (grades 4 and 6). However, it was unknown whether the variance could be explained by the grade group. Based on the reading stage statement (Chall, 1996) and previous reading ability development research (e.g., Mol and Bus, 2011), this study regarded grades 1–3 of primary school as group L; grades 4–6 as group H; and grades 7–12 as grade S. This was informed by García and Cain’s (2014) study which showed that readers had an independent reading ability development period in secondary school, and regarded university learning as grade U, respectively.

#### Language type

Learning cognitive condition statement suggested that the grammatical effect on text comprehension was involvement in noticing and processing the discourse structures (Tagarelli et al., 2011). Due to the familiarity effect on text scripts, more contribution of grammatical knowledge on text comprehension might be found in L2 than in L1 text comprehension tasks (Koda, 1992; Waltzman and Cairns, 2000). The language type might be an explanation of the various empirical correlation findings between reading comprehension and grammatical knowledge.

### Past relevant meta-analytic review

In the past two decades, a few studies (e.g., Jeon and Yamashita, 2014) reviewed the contribution of grammatical knowledge to reading comprehension. For example, Rodd et al. (2015) used a meta-analytic approach to report the effects of localizing semantic grammatical knowledge and written comprehension on brain function. Jeon and Yamashita (2014) reported the correlation between grammatical knowledge and general L2 acquisition. However, past meta-analysis studies failed to solve the significant heterogeneity problem through the moderator analysis, which means that the theoretical contribution to the correlation between reading comprehension and grammatical knowledge requires further investigation. In addition, previous studies only selected a small number of empirical studies for correlation calculation, meaning that the selected studies were not representative. The current picture of the interaction effect with reading stage development and learning cognitive condition statement thus remains unclear.

### The current study

This article extends the current literature concerning the influence of the developmental relations of grammatical knowledge on reading comprehension, investigating the overall correlation between grammatical knowledge and reading comprehension from the recent research published in the past 20 years. This study further examines the interaction effects of the reading stage and learning cognitive condition on the correlation between reading comprehension and grammatical knowledge.

## Method

The official guideline of the meta-analytic approach The Preferred Reporting Items for Systematic reviews and Meta–Analyses (*PRISMA*) was applied to this study, including the literature base, inclusion criteria, coding process, and meta-analytic procedure. The *PRISMA* is an official meta-analytic procedure guideline on meta-analysis implementation.

### Literature base

To avoid the comprehension system mistake (García and Cain, 2014), this study only selected materials written in Chinese and English. The Chinese materials were selected from the CNKI database, which included almost all possible academic resources written in Chinese, and the English materials were searched from Google Scholar, PsycINFO, ERIC, and Pre-Quest. Relevant studies were identified through two groups of keywords; the first group of words was related to grammatical knowledge (grammar*, grammatical*, syntax*, syntactic*, oral cloze test*, sentence completion*, error recognition*, implicit knowledge*, and explicit knowledge*) and the second group of words was related to reading comprehension (sentence comprehension*, paragraph comprehension*, passage comprehension*, text comprehension*, reading comprehension*, reading ability*, comprehension ability*, reading acquisition*, reading performance*, comprehension ability*, and comprehension performance*). This study attempted to select all possible materials with publication dates from 1 January 1998 to 1 December 2022, including published or unpublished research articles, dissertations, and conference articles. Thus, 1,104 articles were selected from the database.

### Inclusion criteria

Materials eligible for inclusion in this meta-analysis were as follows: (a) empirical studies with a minimum of 30 participants, (b) the selected studies should have correlation scores on the correlation between grammatical knowledge and reading comprehension, (c) the participants should be students from grade 1 of primary school to undergraduate students, (d) students were not diagnosed with deaf or blind, (e) studies investigated the concurrent correlation between grammatical knowledge and reading comprehension, (f) studies should provide enough indicators which could be transformed into Fisher’s *z*-transformation, and (g) grammatical knowledge and reading comprehension should come from the same scripts (e.g., L1 grammatical knowledge and L1 reading comprehension, L2 grammatical knowledge and L2 reading comprehension). If the grammatical knowledge and reading comprehension came from different scripts (e.g., L1 grammatical knowledge and L2 reading comprehension), the related study was removed. Thus, 128 articles fulfilled the inclusion criteria.

### Coding process

Studies were coded according to the characteristics of the participants and the measurements by two independent coders. Coding was based on (a) study number, (b) first author’s name, (c) publication year, (d) material type (journal article or dissertation), (e) sampling area, (f) sample size, (g) grade group (L refers to grades 1–3 of primary school, H refers to grades 4–6 of primary school, S refers to secondary school, U refers to university), (h) target language scripts, and (i) correlation effect size. Any unclear information was emailed to the article’s author for clarification. Because this study did not investigate the transfer effect in grammatical knowledge and attempt to examine the hypothesis from the learning cognitive condition theory, the two coders were asked to remove those articles in which the grammatical knowledge and reading comprehension did not come from the same scripts, for example, the correlation between L1 grammatical knowledge and L2 reading comprehension. If one article provided more than one correlation that came from different grade groups, this study treated them as an independent study (Mol and Bus, 2011). If one article provided more than one available correlation indicator in the same grade group, first, this study preferred to select the correlation indicator from the standardized measurements than from the research-developed test. After this step, if the article still had more than one available indicator, Hedges et al. (2010) cluster regression was applied to calculate the final Fisher’s *z*-transformation for this study, ensuring that each study only provided one correlation indicator for further meta-analysis (Mol and Bus, 2011). The internal reliability for two independent coders was 0.96 in the first round of coding. The difference came from sampling area coding; however, this problem was solved by using the country’s name as the sampling area. Thus, 65 articles remained after the coding. Detailed information can be found in Table 1. All materials selected for the meta-analysis have been listed in references with “*.”

**Table 1:** Moderators and outcomes for the matched set of self-report studies in meta-analysis.

Study No	Materials type	First author	Publication time	Sample size	Area	Grade group^a^	Language type	Fisher’s *z*	SE
1	Journal Article	Roth	2002	66	USA	L	L1	0.36	0.13
2	Journal Article	Catts	2002	268	USA	L	L1	0.32	0.06
3	Journal Article	Chamberlain	2008	31	Canada	U	L1	0.79	0.19
4	Journal Article	Gottardo	2009	79	Mexical	L	L2	0.42	0.12
5	Journal Article	Kim	2011	242	USA	L	L1	0.45	0.07
6a	Journal Article	Vellutino	2007	297	USA	L	L1	0.34	0.06
6b	Journal Article	Vellutino	2007	171	USA	H	L1	0.35	0.08
7	Journal Article	Chung	2013	78	HK	S	L1	0.51	0.12
8a	Journal Article	Geva	2012	390	Canada	S	L2	0.66	0.05
8b	Journal Article	Geva	2012	149	Canada	S	L1	0.68	0.08
9	Book Chapter	Rescorla	2000	22	USA	S	L1	0.58	0.23
10	Journal Article	Aryadoust	2016	825	Iran	U	L2	0.65	0.04
11a	Journal Article	Cutting	2009	56	USA	S	L2	0.58	0.14
11b	Journal Article	Cutting	2009	56	USA	S	L2	0.60	0.14
12	Journal Article	Nassaji	1999	60	Canada	U	L2	0.56	0.13
13a	Dissertation	Brimo	2011	193	USA	S	L2	0.42	0.07
13b	Dissertation	Brimo	2011	193	USA	S	L2	0.24	0.07
14	Journal Article	Leider	2013	51	USA	H	L2	0.57	0.14
15	Journal Article	O’Connor	2004	72	USA	S	L1	0.60	0.12
16	Journal Article	Bowey	2005	97	USA	L	L1	0.41	0.10
17a	Journal Article	Farnia	2013	400	Canada	L	L2	0.24	0.05
17b	Journal Article	Farnia	2013	400	Canada	H	L2	0.44	0.05
17c	Journal Article	Farnia	2013	153	Canada	L	L1	0.37	0.08
17d	Journal Article	Farnia	2013	153	Canada	H	L1	0.60	0.08
18	Journal Article	Whyte	2013	26	USA	H	L1	0.54	0.21
19	Journal Article	Lesaux	2006	480	Canada	H	L2	0.50	0.05
20a	Journal Article	Schatschneider	2004	384	USA	L	L1	0.32	0.05
20b	Journal Article	Schatschneider	2004	189	USA	L	L1	0.21	0.07
21	Journal Article	Adlof	2010	433	USA	S	L1	0.62	0.05
22a	Journal Article	Rescorla	2002	34	USA	L	L1	0.34	0.18
22b	Journal Article	Rescorla	2002	34	USA	L	L1	0.34	0.18
22c	Journal Article	Rescorla	2002	34	USA	H	L1	0.34	0.18
22d	Journal Article	Rescorla	2002	34	USA	H	L1	0.60	0.18
23	Journal Article	Silverman	2015	377	USA	H	L1	0.48	0.05
24a	Journal Article	Van Gelderen	2004	397	Netherlands	S	L1	0.62	0.05
24b	Journal Article	Van Gelderen	2004	397	Netherlands	S	L2	0.59	0.05
25	Journal Article	Potocki	2013	131	France	L	L1	0.37	0.09
26	Journal Article	Chaney	1998	41	USA	L	L1	0.34	0.16
27a	Journal Article	Kim	2015	200	Korea	S	L2	0.65	0.07
27b	Journal Article	Kim	2015	200	Korea	S	L2	0.66	0.07
28a	Journal Article	van Gelderen	2003	397	Netherlands	S	L1	0.56	0.05
28b	Journal Article	van Gelderen	2003	397	Netherlands	S	L2	0.56	0.05
29	Journal Article	Chik	2012	274	HK	L	L1	0.54	0.06
30	Journal Article	Oakhill	2003	102	UK	L	L1	0.42	0.10
30	Journal Article	Oakhill	2003	102	UK	S	L1	0.58	0.10
31	Journal Article	Lasagabaster	2001	126	Spain	H	L2	0.58	0.09
32	Journal Article	Jafari	2016	50	Iran	U	L2	0.80	0.15
33a	Journal Article	Oakhill	2012	102	UK	H	L1	0.42	0.10
33b	Journal Article	Oakhill	2012	92	UK	H	L1	0.58	0.11
33c	Journal Article	Oakhill	2012	83	UK	H	L1	0.51	0.11
34	Journal Article	Goff	2005	180	Australia	H	L1	0.65	0.08
35a	Journal Article	Shiotsu	2007	107	UK	U	L1	0.73	0.10
35b	Journal Article	Shiotsu	2007	182	UK	U	L2	0.71	0.08
35c	Journal Article	Shiotsu	2007	591	UK + Japan	U	L2	0.73	0.04
36a	Journal Article	Proctor	2011	294	USA	H	L1	0.45	0.06
36b	Journal Article	Proctor	2011	294	USA	H	L1	0.55	0.06
37	Journal Article	Foorman	2015	218	USA	L	L1	0.44	0.07
38	Journal Article	Park	2012	28	Korea	U	L2	0.81	0.20
39	Journal Article	Zhang	2012	190	China	U	L2	0.22	0.07
40	Journal Article	Mokhtari	2012	32	USA	H	L1	0.52	0.19
41	Journal Article	Gui	2018	181	China	U	L2	0.60	0.08
42	Journal Article	Xiang	2016	168	China	S	L2	0.59	0.08
43a	Journal Article	Gong	2009	68	China	S	L2	0.68	0.12
43b	Journal Article	Gong	2009	68	China	S	L2	0.56	0.12
44	Journal Article	Lu	2015	106	China	S	L2	0.69	0.10
45	Journal Article	Wu	2016	70	Japan	U	L2	0.66	0.12
46	Journal Article	Shen	2014	68	China	U	L2	0.68	0.12
47	Dissertation	Liao	2012	44	China	S	L2	0.50	0.16
48	Dissertation	Zhang	2011	31	China	U	L2	0.84	0.19
49a	Dissertation	Luo	2010	63	China	U	L2	0.71	0.13
49b	Dissertation	Luo	2010	63	China	U	L2	0.72	0.13
50	Dissertation	Tang	2013	188	China	S	L2	0.60	0.07
51a	Dissertation	Deng	2014	35	China	U	L2	0.62	0.18
51b	Dissertation	Deng	2014	35	China	U	L2	0.74	0.18
52	Journal Article	Shen	2011	68	China	U	L2	0.81	0.12
53a	Journal Article	Chen	2008	58	China	L	L1	0.40	0.14
53b	Journal Article	Chen	2008	58	China	L	L1	0.31	0.14
54	Journal Article	Li	2008	50	China	U	L2	0.79	0.15
55	Dissertation	Wang	2012	110	China	S	L2	0.48	0.10
56	Journal Article	Zhang	2011	190	China	U	L2	0.22	0.07
57	Journal Article	Gong	2010	72	China	S	L2	0.66	0.12
58	Dissertation	Yan	2012	91	China	U	L2	0.60	0.11
59	Journal Article	Li	2016	199	China	L	L1	0.34	0.07
60a	Dissertation	Jiang	2003	188	China	S	L2	0.61	0.07
60b	Dissertation	Jiang	2003	188	China	S	L2	0.60	0.07
61	Journal Article	Zhang	2017	77	China	U	L2	0.83	0.12
62	Journal Article	Guan	2007	188	China	S	L2	0.60	0.07
63	Journal Article	Chen	2005	41	China	U	L2	0.57	0.16
64a	Dissertation	Wang	2001	176	China	S	L2	0.56	0.08
64b	Dissertation	Wang	2001	176	China	S	L2	0.60	0.08

a L = lower grade, from kindergarten to grade 2, H = higher grade, from grade 3 to grade 6, S = secondary school, grade 7 to grade 12. U = undergraduate students and master students.

### Meta-analytic procedure

This study removed two articles conducted by Zhang (2011, 2012), and one study conducted by Brimo et al. (2017) due to the effect size, which was over 3.5 standard deviation (García and Cain, 2014). As a result, 62 articles with 86 studies were included in the final analysis. The researchers input the correlation indicator through comprehension meta-analysis 3.0 and transformed it into Fisher’s *z* for further analysis. This study selected Fisher’s *z*-transformation because *z* is approximately constant, and *z* has asymmetrical distribution (Borenstein et al., 2010). The values of Fisher’s *z* were 0.10 (*r* = 0.10), 0.31 (*r* = 0.30), and 0.50 (*r* = 0.50), denoting small effect size, moderate effect size, and large effect size, respectively (Borenstein et al., 2010).

This study used the random-effects model to report the effect size (Borenstein et al., 2010) and also reported a 95% confidence interval (*CI*). The effect size was interpreted as significant if the *CI* did not include zero. Next, the *Q* value was reported to examine the heterogeneity within materials. If the *Q* value reached a significant level (*p* < 0.05), then a meta-regression analysis was carried out to further analyze the effects of selected moderators (Hedges and Pigott, 2004; Borenstein et al., 2010). To compare the effect sizes across factors, this study applied the following equation to examine the difference of *Teta* (Durlak, 2009; Lenz, 2013): *Teta* = Diff / SE, Diff = *z*_1_ – *z*_2_, SE = sqrt (Variance *z*_1_ + Variance *z*_2_).

If |*Teta*| ≥ 2.58, the factor difference was interpreted as significant (*p* < 0.01). To examine publication bias, this study examined Rosenthal’s fail-safe number, a funnel plot through the trim-and-fill approach, the rank correlation test, and Egger’s regression test (Borenstein et al., 2010).

## Results

### Descriptive statistics

A total of 62 articles with 88 studies (*N* = 14,852) were included in this meta-analysis. In total, 16 studies were master’s dissertations and doctoral theses, and the other 72 studies were published in peer-reviewed journals. Specifically, 20 studies (*n* = 3,324) investigated the correlation between grammatical knowledge and reading comprehension in group L, 16 studies (*n* = 2,749) reported the correlation in group H, 29 studies (*n* = 5,652) investigated the correlation in secondary school students (group S), and 21 studies (*n* = 2,747) examined the correlation in higher education students (group U). For language type, 41 studies (*n* = 6,505) investigated the correlation between grammatical knowledge and reading comprehension in L1 scripts, and 47 studies (*n* = 8,347) focused on the relationship between L2 grammatical knowledge and L2 reading comprehension.

### Meta-analysis

**Figure 1:**
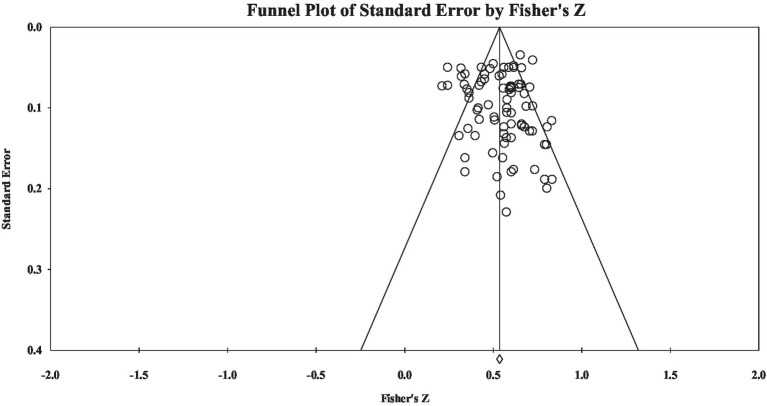
A funnel plot of overall effect size.

Table 2 provides the results of the correlation effect size between grammatical knowledge and reading comprehension. The overall effect size was large (Fisher’s *z* = 0.54); however, the heterogeneity analysis showed that the materials’ heterogeneity was significant (*Q* = 271.48, *p* < 0.001). Moderator analysis through meta-regression showed that grade group explained 59% variance of materials’ heterogeneity and language type explained 13% variance of materials’ heterogeneity. Publication bias examination showed the effect size-distributed symmetry (see Figure 1): Rosenthal’s fail-safe number was 8,824, the tau value in the rank correlation test was insignificant (tau = 0.04, *p* > 0.05), and the intercept value in Egger’s regression test was not significant (intercept = 0.26, *p* > 0.05). The publication bias examination showed that the current study did not have significant publication bias.

**Table 2:** Correlation of overall effect between grammatical knowledge and reading comprehension with subgroup analysis.

Variable	*k*	Fisher’s z	Variance	95% *CI*	*Q*	*N* fail-safe	*Teta* (between grade group)
Overall	86	0.54	0.0003	[0.51, 0.57]	271.48^***^	855	*Teta* (H & L) = 4.60, *Teta* (H &S) = 4.90, *Teta* (S & U) = 3.27. *Teta* (L & S) = 10.21, *Teta* (L & U) = 11.67, *Teta* (H & U) = 7.07
Group L	20	0.36	0.0004	[0.32, 0.40]	23.29	124	
Group H	16	0.49	0.0004	[0.45, 0.53]	11.52	142	
Group S	29	0.61	0.0002	[0.58, 0.63]	9.81	323	
Group U	21	0.69	0.0004	[0.65, 0.73]	10.77	269	

^***^p < 0.001.

**Figure 2:**
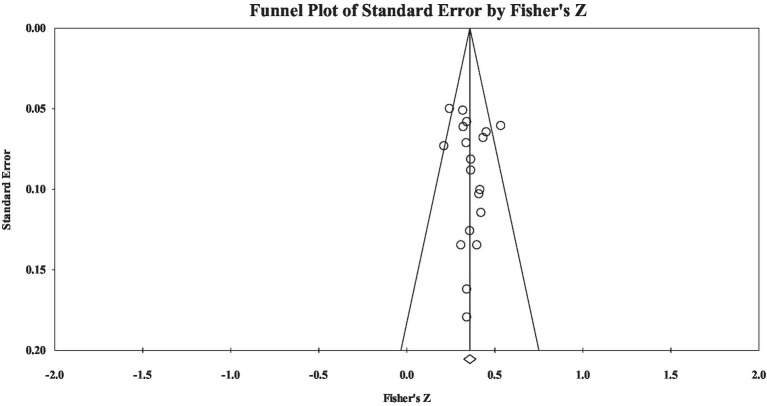
A funnel plot of group L effect size.

Because the majority of materials’ heterogeneity resulted from the grade group, this study further investigated the correlation effect size between grammatical knowledge and reading comprehension in four grade groups. Regarding group L, the results showed that the correlation effect size was moderate (Fisher’s *z* = 0.36). The heterogeneity analysis showed that the materials’ heterogeneity was not significant (*Q* = 23.29, *p* > 0.05). Publication bias examination showed the effect size-distributed symmetry (see Figure 2): Rosenthal’s fail-safe number was 1,935, the tau value in rank correlation test was insignificant (tau = 0.06, *p* > 0.05), and the intercept value in Egger’s regression test was not significant (intercept = 0.41, *p* > 0.05). the results showed that the current study did not have significant publication bias.

**Figure 3:**
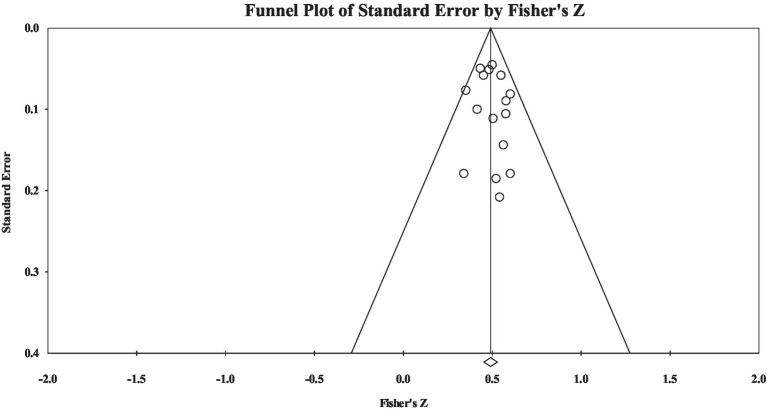
A funnel plot of group H effect size.

Regarding group H, the results showed the correlation effect size was nearly large (Fisher’s *z* = 0.49), and heterogeneity analysis showed that materials’ heterogeneity was not significant (*Q* = 11.52, *p* > 0.05). Publication bias examination showed the effect size-distributed symmetry (see Figure 3): Rosenthal’s fail-safe number was 2,255, the tau value in rank correlation test was insignificant (tau = 0.03, *p* > 0.05), and the intercept value in Egger’s regression test was not significant (intercept = 0.37, *p* > 0.05). The results showed that the current study did not have significant publication bias.

**Figure 4:**
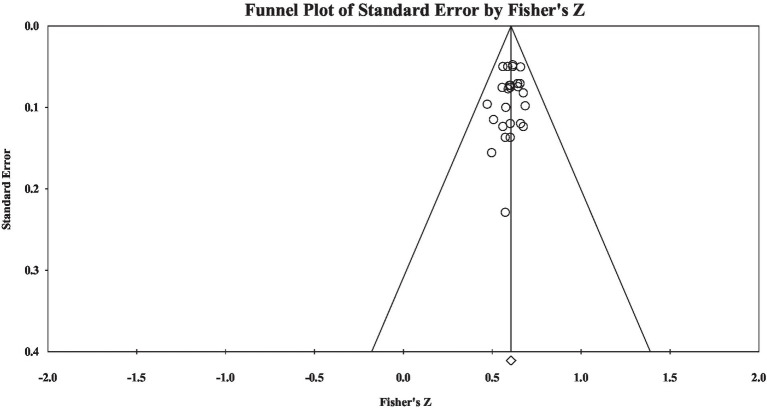
A funnel plot of group S effect size.

Regarding group S, the results showed that the correlation effect size was large (Fisher’s *z* = 0.61), and heterogeneity analysis showed that materials’ heterogeneity was not significant (*Q* = 9.81, *p* > 0.05). Publication bias examination showed the effect size-distributed symmetry (see Figure 4): Rosenthal’s fail-safe number was 2,555, the tau value in rank correlation test was insignificant (tau = −0.09, *p* > 0.05), and the intercept value in Egger’s regression test was not significant (intercept = −0.16, *p* > 0.05). The results showed that the current study did not have significant publication bias.

**Figure 5:**
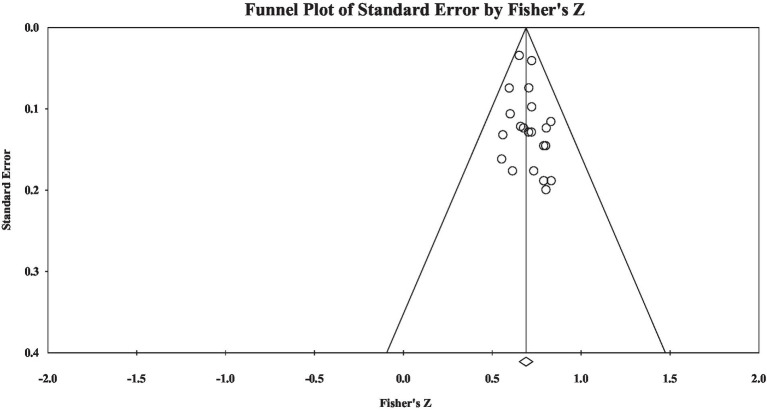
A funnel plot of group U effect size.

Regarding group U, the results showed the correlation effect size was large (Fisher’s *z* = 0.69), and heterogeneity analysis showed that materials’ heterogeneity was not significant (*Q* = 10.77, *p* > 0.05). Publication bias examination showed the effect size-distributed symmetry (see Figure 5): Rosenthal’s fail-safe number was 5,188, the tau value in rank correlation test was insignificant (tau = 0.14, *p* > 0.05), and the intercept value in Egger’s regression test was not significant (intercept = 0.36, *p* > 0.05). The results showed that the current study did not have significant publication bias.

Effect size comparison examination showed that the difference between each of the two groups’ effect size was significant (*Teta*_H & L_ = 4.60, *p* < 0.01; *Teta*_H &S_ = 4.90, *p* < 0.01; *Teta*_S & U_ = 3.27, *p* < 0.01).

## Discussion

**Figure 6:**
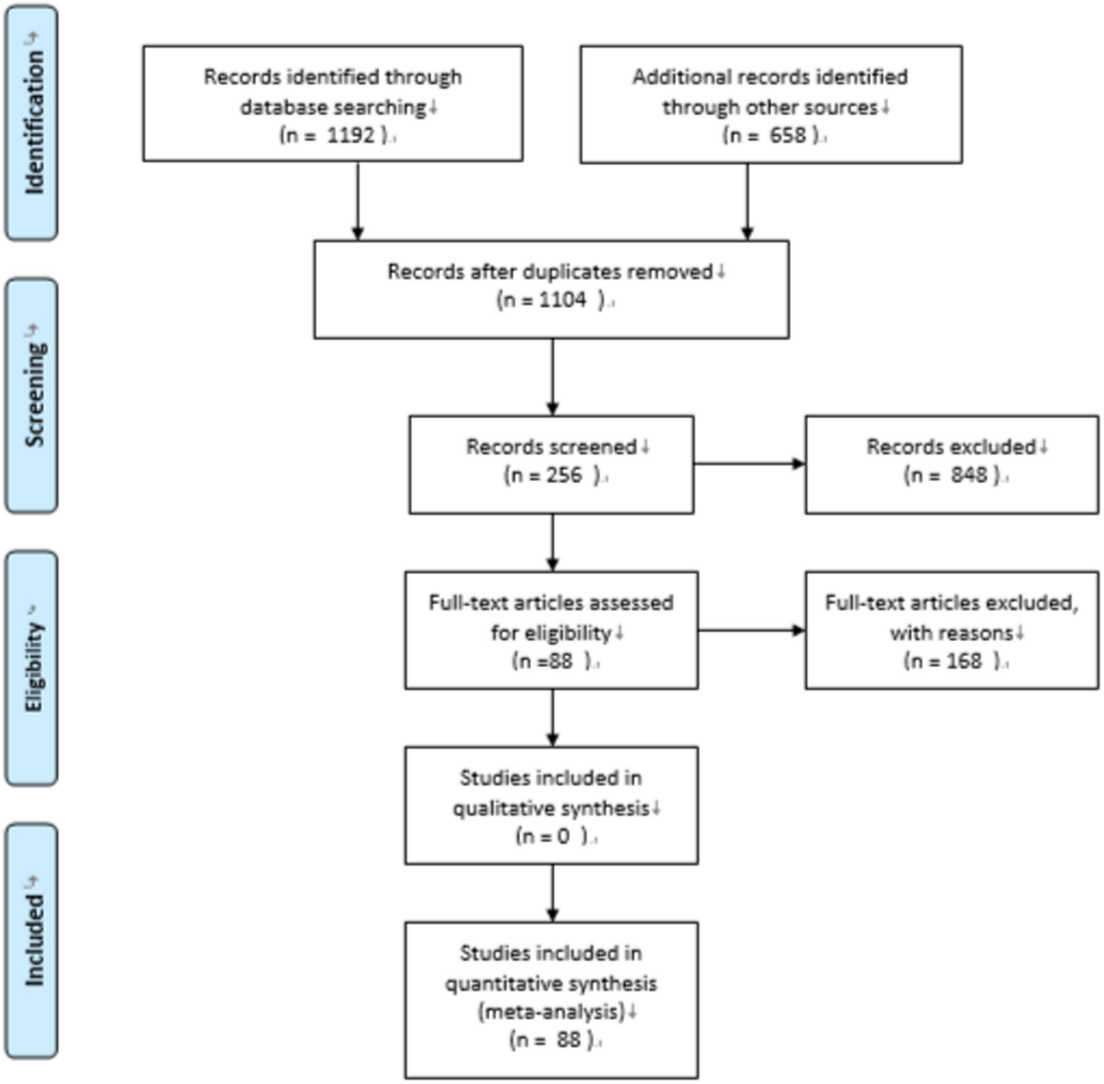
A flow diagram for materials search.

The current study showed that the overall correlation effect size was large between grammatical knowledge and reading comprehension. Effect size increased significantly from the lower grade group to the higher grade group and from moderate to large. The results indicated that the cohesive tie on text comprehension interacted significantly with the reading stage. At each reading stage, the interaction effect of learning cognitive condition was not significant on the correlation between reading comprehension and grammatical knowledge (Figure 6).

### Grammatical knowledge on reading comprehension

A large effect size was found in the overall correlation between grammatical knowledge and reading comprehension. Jeon and Yamashita (2014) reported that the correlation between grammatical knowledge and reading comprehension in L2 scripts was extremely high (*r* = 0.85) in groups S and U, which was very different from the current results. Jeon and Yamashita (2014) had some obvious limitations. First, they only included 18 articles in their meta-analysis, and the results showed that the *Q* value from heterogeneity analysis was significant. Second, Jeon and Yamashita (2014) did not remove over 3.5 standard deviation effect sizes as the outlier, which showed that the correlation was unclear and did not generate convincing conclusions due to the significant heterogeneity problem. Through the literature search, the current results were consistent with most empirical studies, which showed that the average correlation between grammatical knowledge and reading comprehension was nearly large (Mecartty, 2000; Gersten et al., 2001; Perfetti et al., 2005), indicating that the grammatical knowledge significantly determined the comprehension process. Moreover, this result informed how the cohesive tie function of grammatical knowledge might have great determining power on reassembling decoded words into phrases and clauses, allowing readers to be more efficient in detecting and correcting reading errors to enhance their comprehension process indirectly (e.g., Slobin, 1966; Bowey, 2005; Kempen et al., 2012).

### Grade group effect

The current results showed that the higher grade group had a significantly larger effect size than the lower grade group. This result was consistent with the majority of past longitudinal studies (e.g., Oakhill et al., 2003; Farnia and Geva, 2013). The reasons for the consistency could be that the requirements, the complicated structure, and the organization level of text comprehension were higher in the higher grade group, resulting in the fact that the requirement of the cohesive tie function was higher in the text coherence clues process (Cain, 2003; Westerveld et al., 2008; Nuske and Bavin, 2015) and organization structure cognition (Oakhill and Cain, 2000; Vaughan and Dillon, 2006; Kendeou et al., 2007). This result informed that the function of the cohesive tie might continue developing after the age of 16, which was different from the majority of comprehension factors, such as decoding ability, vocabulary knowledge, and metalinguistic knowledge (Mol and Bus, 2011; García and Cain, 2014; Trapman et al., 2014). The current result also echoed reading stage theory regarding grammatical knowledge function on text reading comprehension, where the more complicated-level text comprehension needed a higher application of grammatical knowledge for coherence inference.

### Language type effect

In each grade group, the language type was not a significant moderator on the correlation between grammatical knowledge and reading comprehension. This result echoed the previous study, which showed that linguistic skills could work similarly across languages’ scripts (Cummins, 1986; Gottardo and Mueller, 2009; Perfetti and Stafura, 2014). In other words, grammatical knowledge could have a transfer effect across all languages, suggesting that grammatical knowledge had an independent cognition function on text comprehension. Readers received text information and delivered target comprehension information into the situation model-building process. The grammatical knowledge worked together with other linguistic skills on text meaning coherence, which indicates that this process encompassed the integrated process and the synthesizing process on word reading and sentence meaning judgment in situation model construction. According to the current results, grammatical knowledge may have an independent effect on reading comprehension across various scripts’ text reading, which means that, for bilingual learners’ reading comprehension, a transfer or compensation effect would help readers to apply either L1 or L2 grammatical knowledge to other language scripts’ comprehension (Sparks et al., 2008). Therefore, readers only need to fully develop grammatical knowledge in one language script, and then, the grammatical knowledge can work in other scripts’ text comprehension processes.

### Limitations

The current study has several limitations. First, this study selected materials that were only written in Chinese or English. Materials written in other languages were not considered. Second, the selected materials only reported the correlation between grammatical knowledge and reading comprehension in Chinese reading, Dutch reading, English reading, and French reading. Next, although past studies mentioned that grammatical knowledge should involve morphological knowledge and verb forms, due to inconclusive findings, this study excluded these two variables. In future studies, if the researcher has an agreed definition in the grammatical knowledge category, it should be further explored. Finally, meta-regression only presented how the extent of the heterogeneity of the materials could be explained by the moderator, while the interaction effect mechanism of each moderator was not identified. For example, this study reported that the grade group and language types were two significant moderators on the overall correlation but did not investigate the internal working interaction effect between these two moderators.

## Conclusion

The study’s conclusions were drawn from the combined results of 86 studies conducted with more than 14,000 readers. The reading comprehension measures only included Chinese, Dutch, English, and French reading comprehension test in the current study’s database. To summarize, this meta-analysis confirmed that the overall correlation between grammatical knowledge and reading comprehension was large (Fisher’s *z* = 0.54). The cohesive tie of the grammatical knowledge on reading comprehension had a significant interaction effect with the reading stage, and the higher requirement of reading comprehension showed higher function application of cohesive tie. The results indicated that the cohesive tie function of grammatical knowledge had a transfer effect across different scripts’ reading comprehension. The correlation pattern between grammatical knowledge and text reading comprehension was similar across Chinese, Dutch, English, and French scripts.

## Author contributions

XM and YD had similar contribution, including data analysis and data collection. All authors contributed to the article and approved the submitted version.

## Funding

This paper received financial support by intramural project of Guangzhou Huashang College (2021HSDS36).

## Conflict of interest

The authors declare that the research was conducted in the absence of any commercial or financial relationships that could be construed as a potential conflict of interest.

## Publisher’s note

All claims expressed in this article are solely those of the authors and do not necessarily represent those of their affiliated organizations, or those of the publisher, the editors and the reviewers. Any product that may be evaluated in this article, or claim that may be made by its manufacturer, is not guaranteed or endorsed by the publisher.
